# Nasal Obstruction and High Mallampati Score as Risk Factors for Obstructive Sleep Apnea

**DOI:** 10.1590/S1808-86942010000500010

**Published:** 2015-10-22

**Authors:** Marcos Marques Rodrigues, Ralph Silveira Dibbern, Carla W. Kruel Goulart

**Affiliations:** 1 Resident Physician - Department of Otorhinolaryngology - Santa Casa de Limeira; 2 MSc in Otorhinolaryngology - USP-Ribeirão Preto, ENT at the Department of Otorhinolaryngology - Santa Casa de Limeira; 3 Resident Physician - Department of Otorhinolaryngology - Santa Casa de Limeira. Irmandade da Santa Casa de Misericórdia de Limeira

**Keywords:** sleep apnea, obstructive, nasal obstruction.

## Abstract

**Abstract:**

Respiratory sleep disorders are strongly associated with upper airway patency. Nasal obstruction is associated with higher incidences of sleep apnea, primarily by increasing the negative pressure on the airway during inspiration.

**Aims:**

To evaluate the influence of nasal obstruction in the worsening of sleep apnea in patients with OSA and a high score on the modified classification of Mallampati.

**Materials and Methods:**

We evaluated and classified 206 patients complaining of snoring, and with a past suggestive of OSA through the Modified Mallampati score, Friedman, nasal obstruction and the severity of OSA by AHI.

**Results:**

168 patients who underwent polysomnography were included. Cross-plotting was made comparing the modified Mallampati score, nasal obstruction and AIH. The odds ratio between high Mallampati score and AHI was OR = 5.053, 95% CI = 1.458 to 7.517 (p = 0.0071). High Mallampati score associated with nasal obstruction was correlated with OSAS (p = 0.0227). However the influence of nasal obstruction on the relationship of high Mallampati score and OSA was not significant: OR = 2.850, 95% CI = 0.992 to 8.189.

**Conclusion:**

The combination of high Mallampati score and nasal obstruction represents a greater risk factor for worsening of OSA.

## INTRODUCTION

Respiratory sleep disorders are associated with upper airway obstruction. Described in 1973 by Cristian Guilleminault^1^, the Sleep Obstructive Apnea Syndrome (SOAS) is a disease characterized by airway obstruction during sleep, causing apnea associated with chest respiratory effort1. Clinical manifestation is variable, more prevalent among obese men with large neck circumference^2^.

A recent epidemiological study carried out in São Paulo - Brazil shows that the prevalence of SOAS is of 32.8% in the adult population of this city. The risk factors associated with the syndrome development were: male gender, Body Mass Index (BMI) > 25kg/m^2^, low socioeconomic status, advanced age and women at menopause. ^3^

Since its discovery, SOAS has been broadly studied and it proved to be a disease which is extremely important and prevalent. It is important because it is brings about relevant morbidity, association with numerous diseases, such as coronary disease^4^, stroke^5^, dyslipidemia and diabetes mellitus^4^. It is prevalent because it affects 32.8% of the population^6^,^3^; however, it is estimated that it is a highly underdiagnosed disease^7^. About 20% of the adult population report snoring and 95% of the patients with SOAS complain of snoring^8^.

Risk factors for sleep apnea and snoring are present in patients between 40 and 65 years, most of them males, obese, smokers, drinkers and sedentary^9^. The main physical findings include an enlargement of the neck circumference, oropharyngeal obstruction, soft palate laxity, nasal obstruction, turbinate hypertrophy, nasal septum deformity, nasal cavity tumors, tonsil hypertrophy, macroglossy, retrognatia and temporomandibular deformities^10^.

It is extremely important to fully assess the upper airways when assessing a patient with SOAS, especially the nasal cavity. Nasal obstruction is associated with an increase in sleep apnea, especially because of an increase in the negative pressure caused to the airways during inspiration^11^.

## OBJECTIVES

To assess the influence of nasal obstruction associated with the modified Mallampati classification in SOAS severity.

## MATERIALS AND METHODS

This study was approved by the Ethics in Research Committee, under protocol # 112/08 and it was duly registered in the Clinical Trials Registry. 206 snoring patients and clinical history suggesting sleep apnea were assessed in the SOAS Ward, with symptoms of daytime sleepiness, unrestorative sleep, nasal obstruction and snoring. The sample had 110 men (53%) and 96 women (47%). All the patients were submitted to an assessment protocol including interview, Epworth's scale, Stanford snoring scale, Friedman's classification and complete ENT exam - including oral exam, patients classification according to the modified Mallampati score and anterior rhinoscopy, later confirmed by nasal fibroscopy, defining septal deviations and inferior turbinate hypertrophy.

The Epworth's scale is broadly used to assess the degree of daytime sleepiness, being made up of eight questions about the person's level of daytime sleepiness in given daily activities. The scale goes from 0 to 24: daytime sleepiness is considered when the score is higher than 10 points^12^. The Friedman scale is used to assess oropharyngeal obstruction evaluating tonsil size by means of the Modified Mallampati Classification and BMI - varying between I and IV^13^.

The patients were submitted to nasofibroscopy with the 3.2mm Machida ENT-30 PIII flexible scope in order to assess the level of upper airway obstruction. Nasal septum deviation and/or inferior turbinate hypertrophy were confirmed by nasal fibroscopy done under a spray of 2% lidocaine in each of the nostrils without vasoconstriction in order not to have a biased evaluation. All patients were offered a 30-day oral antihistaminic treatment and topical nasal steroid for 03 months.

Afterwards, all the patients were referred for nocturnal polysomnography at the sleep laboratory, with full monitoring of the following parameters: electro-oculogram, leg electromyography, air flow, chest and abdominal movement, EEG, EKG, heart rate and oxygen saturation. The patients were distributed according to disease severity and according to the criteria established by the American Sleep Academy Task-Force^1^.

### Exclusion Criteria

Patients with the following conditions were taken off this study:

• Morbid obesity (BMI>40);
• Craniofacial abnormalities: cranio-dysostosis, craniostenosis and meningomyelocele;
• Nasal obstruction due to polyposis;
• Nasal obstruction due to nasal tumors;

### Statistics

Of the 206 patients assessed according to the protocol, 168 patients were included. The remaining patients matched 1 or more exclusion criteria.

Based on the Sleep Hypopnea-Apnea Index (HAI), considered the outcome variable of the model, the valid sample was later broken down into two groups: Group I with patients with primary snoring or mild SOAS (HAI ≤ 15 events/hour) and Group II with moderate to severe SOAS patients (HAI > 15 events/hour). We first selected the variables and, based on the results, we selected the following variables:

1 Modified Mallampati classification with the same criteria used in the Friedman classification^14^. The patients were broken down into two groups: patients with low Mallampati score (1-2) and those with high Mallampati score (3-4).
2 As to the presence of nasal obstruction, the patients were broken down into two groups. We included those patients with nasal septum deviation and/or inferior turbinate hypertrophy, after the treatment of the nasal atopy described above. Therefore, patients with nasal polyps and those with nasal tumors were excluded.

In order to analyze the data, we used the logistic function (PROC LOGISTIC) from the SAS software, as suggested by Allison^14^. We also used the SAS/STAT function (SAS INSTITUTE, 2001), in accordance with the routine presented by Stokes^15^. The model adjustment was assessed by the AIC and -2 Log L criteria, and the statistical significance evaluation of the estimated parameters was carried out by the maximum similarity ratio. We included in the study the interaction effect between the Mallampati variables and Nasal Obstruction - for it proved significant - in an attempt to understand its behavior.

## RESULTS

Table 2 shows the analysis of the effects studied based on the Chi-square significance test. We associated the variable studied with the HAI higher than 15 events/hour.

**Table 2:** SOAS Protocol Variables in relation to a HAI >15 events/hour.

Variable	p (Chi-square Test)
High Mallampati	0,0071(s)
BMIa > 25kg/m2	0,0043(s)
Epworth > 10 pts	0,0064(s)
Snoring	0,4565(ns)
Nasal Obstruction	0,6670(ns)
High Mallampati associated with Nasal Obstruction	0,0227(s)
Male Gender	0,0021(s)

a Body Mass Index

The Mallampati modified classification is positively associated with the HAI (p = 0.0071). The nasal obstruction alone is not associated with the HAI (p= 0.6670). When analyzed as factors associated with the High Mallampati score and Nasal Obstruction, they were positively associated with the HAI (p=0.0227) Table 1.

**Table 1:** Distribution of the Variables in the SOAS Protocol.

	STANFORD	BMIa	EPWORTH	AGE
Mean	8.16	29.42	11.25	47.95
Standard Deviation	2.10	5.55	5.14	11.25
Minimum	2	16.44	0	17
Maximum	10	51.02	24	77

^a^ Body Mass Index

As far as the other variables analyzed are concerned, BMI, Epworth's Scale and Male gender were statistically associated with the HAI. Since almost the entire sample complained of snoring, such variable was not positively associated with the HAI.

Since the Mallampati and nasal obstruction interaction was significant (p<0.05), we analyzed how the Mallampati score interacted with Nasal Obstruction. Thus, we unfolded the interaction, adjusting a model with the Mallampati effect within each Nasal Obstruction level and a model with the Nasal Obstruction variable within each Mallampati level. The results are presented on Table 3.

**Table 3:** Interaction of the different levels of the Mallampati score and Nasal Obstruction with an HAI > 15 events/hour.

Mallampati	Nasal Obstruction	OR	CI 95%
Low	No	1.00	-
Low	Yes	2.270	0.728 - 7.076(ns)
High	Yes	2.850	0.992 - 8.189(ns)

OR: “Odds Ratio”

CI 95%: Confidence Interval

This table shows the Mallampati and Nasal Obstruction interaction, and the analysis had HAI > 15 events/hour as dependant variable. The risk of a patient having severe SOAS is greater when there is High Mallampati in the absence of Nasal Obstruction (OR=5.053, CI 95%=1.458 - 7.517). When we assessed the presence of high Mallampati and Nasal Obstruction, the OR was 2.850 (CI 95% = 0.992 - 8.189) and 2.27 (CC 95% = 0.728 - 7.076) with mild Mallampati and nasal obstruction.

There was no statistical significance in the analysis of the nasal obstruction influence on the Mallampati score correlation, in other words, nasal obstruction does not interfere in the interaction between the Mallampati score and the HAI > 15 events/hour; nonetheless, the association of both variables was positive (p=0.0227).

## DISCUSSION

SOAS is a disease which mainly affects the upper airways (UAW), and it can be caused by obstruction of numerous structures such as the palate, nasal septum deviation, elongated uvula, palatine tonsil, macroglossy and posterior pharyngeal wall. Each point can have a different impact on the UAW obstruction and in different degrees in different patients10. It is an important disease in the current medical setting because of its association of high incidence and comorbidity^3^.

In our sample, the male gender was positively correlated with the presence of SOAS, corroborating numerous populational studies carried out in Brazil^3^, the United States and Europe^2^. Men tend to be more obese, have a thicker neck and a greater likelihood of having UAW obstruction during sleep.

Obesity represented by a BMI > 25Kg/m^2^ is associated with SOAS in different levels. The higher the BMI, the greater the HAI tended to be in our sample. By the same token, the Epworth Sleepiness Scale is significantly correlated with the HAI, showing that excess day-time sleepiness is an important symptom and must be extensively evaluated in SOAS predisposition assessment.

The influence of the high Mallampati score on sleep respiratory disorders, especially SOAS, is very important and it is described in numerous studies^17^. Patients with a high Mallampati score tend to have obstruction, especially because of macroglossia, precluding air passage from the nose and mouth to the lower airways (LAW).

By the same token, the association of SOAS and nasal obstruction has been broadly studied in the last two decades^18^. Udaka et al.^19^ showed, by means of a populational study with 4,818 patients, a positive correlation between nasal obstruction, daytime sleepiness and sleep apnea. Patients with nasal obstruction tend to have greater negative pressure, thus causing UAW obstruction and consequently oxygen desaturation and increase in chest stress^10^. In our series, the presence of nasal obstruction alone was not statistically associated with SOAS.

The presence of a high Mallampati score associated with nasal obstruction increases the likelihood of the patient developing apnea and its consequent worsening. Patients with such conditions have two important obstruction points, increasing SOAS severity because the patient would put up a greater chest stress in order to overcome two levels of obstruction^18^.

We observed that in a series of patients with High Mallampati score, that it is an important isolated risk factor for apnea appearance and worsening.

The association between a high Mallampati score and Nasal Obstruction is a risk factor for the development and worsening of apnea. The two variables, when associated, are statistically correlated with the apnea severity. However, nasal obstruction does not impact on the correlation between a high Mallampati score and HAI, therefore not causing additional risk to the worsening of the apnea in this subpopulation.

Surgical planning and the careful assessment of SOAS patients are fundamental in order to establish the different obstructive anatomical sites. Patients with high Mallampati score tend to have a high Friedman score and a lower uvulopalatopharyngoplasty success^13^. Patients with such condition associated with nasal obstruction must be better assessed, since one can reduce the severity of apnea with the correction of the nasal obstruction, either clinically or surgically.

In the cases of nasal surgery in apneic patients, in our service we prefer the conventional nasal septum surgery and partial turbinectomy without the use of nasal packing, for having less comorbidity and lower immediate post-operative complication rates, which is usually critical to these patients. There are reports of the use of turbinectomy by radiofrequency with lower postoperative morbidity, less edema and lower risks of bleeding and sinechia^20^.

## CONCLUSION

The high Mallampati score is an important isolated risk factor for the worsening of the apnea. Nasal Obstruction becomes an important additional risk factor when associated to apneic patients with a high Mallampati score. Nasal obstruction alone is not associated with SOAS. Nonetheless, we stress the relevance of a careful assessment of the UAW and its possible points of obstruction in the evaluation and surgical planning of patients with sleep apnea.

## References

[1] Sleep-related breathing disorders in adults: recommendations for syndrome definition and measurement techniques in clinical research. The Report of an American Academy of Sleep Medicine Task Force. Sleep. 1999 Aug 1;22(5):667-89. Review. 10450601

[2] Stradling JR, Crosby JH (1991). Predictors and prevalence of obstructive sleep apnoea and snoring in 1001 middle aged men. Thorax..

[3] Tufik S, Santos-Silva R, Taddei JA, Bittencourt LRA (2010). Obstructive Sleep Apnea Syndrome in the Sao Paulo Epidemiologic Sleep Study. Sleep Med..

[4] Peppard PE, Young T, Palta M, Skatrud J (2000). Prospective study of the association between sleep-disordered breathing and hypertension. N Engl J Med..

[5] Yaggi H.K., Concato J., Kernan W.N., Lichtman J.H., Brass L.M., Mohsenin V (2005). Obstructive Sleep Apnea as a Risk Factor for Stroke and Death. Engl J Med..

[6] Davies RJO, Stradling JR (1996). The epidemiology of sleep apnoea. Thorax..

[7] Lindberg E, Gislason T (2000). Epidemiology of sleep-related obstructive breathing. Sleep Med Rev..

[8] Jennum P, Sjol A (1992). Epidemiology of snoring and obstructive sleep apnea in a Danish population, age 30-60. Sleep Res..

[9] Partinen M, Telakivi T (1992). Epidemiology of obstructive sleep apnea syndrome. Sleep..

[10] Chervin RD, Guilleminault C (1996). Obstructive sleep apnea and related disorders. Neurol Clin..

[11] Atkins M, Taskar V, ClaytonN Stone P, Woodcock A (1994). Nasal resistance in obstructive sleep apnea. Chest..

[12] Johns M.W (1991). A new method for measuring daytime sleepiness: The Epworth sleepiness scale. Sleep..

[13] Friedman M, Vidyasagar R, Bliznikas D, Joseph N (2005). Does Severity of Obstructive Sleep Apnea-Hipopnea syndrome predict Uvulopalatopharyngoplasty outcome?. Laryngoscope..

[14] Allison P.D. (1999). Logistic regression using the SAS® System: theory e application..

[15] Stokes M.E., Davis C.S., Koch G.G. (1995). Categorical data analysis using the SAS® System..

[17] Friedman M, Tanyeri H, LaRosa M, Landsberg R, Vaidyanathan K, Pieri S (1999). Clinical predictors of obstructive sleep apnea. Laryngoscope..

[18] Liistro G., Rombaux Ph., Belge C., Dury M., Aubert G., Rodenstein D.O (2003). High Mallampati score and nasal obstruction are associated risk factors for obstructive sleep apnoea. Eur Respir J..

[19] Udaka T, Suzuki H, Fujimura T, Hiraki N, Shiomori T, Kitamura T (2007). Relationships between nasal obstruction, observed apnea, and daytime sleepiness. Otolaryngology-Head and Neck Surgery..

[20] Kezirian EJ, Powell NB, Riley RW, Hester JE (2005). Incidence of Complications in Radiofrequency Treatment of the Upper Airway. Laryngoscope..

